# Enhancement of Arc Erosion Resistance in AgCuO Electrical Contact Materials through Rare Earth Element Doping: First-Principles and Experimental Studies

**DOI:** 10.3390/ijms241612627

**Published:** 2023-08-10

**Authors:** Haitao Wang, Yanling Wang, Jingqin Wang, Qinglong Cai, Dekao Hu

**Affiliations:** State Key Laboratory of Reliability and Intelligence of Electrical Equipment, Electrical Engineering Department, Hebei University of Technology, Tianjin 300401, China; wangyl303@163.com (Y.W.); jqwang@hebut.edu.cn (J.W.); 202131404105@stu.hebut.edu.cn (Q.C.); hudekao@163.com (D.H.)

**Keywords:** first-principles calculations, AgCuO electrical contact materials, arc erosion, material transfer, surface profile, rare earth

## Abstract

To investigate the stability and electrical and physical properties of undoped CuO and CuO doped with rare earth elements, electronic structures and elastic constants were calculated using first-principles density functional theory. Additionally, experimental verification was carried out on AgCuO and AgCuO-X (La, Ce, Y) electrical contacts, which were prepared using sol–gel and powder metallurgy methods. The contacts were tested under an 18 V/15 A DC resistive load using the JF04D contact material testing system. Arc parameters were analyzed, and three-dimensional surface profilometry and scanning electron microscopy were used to study the altered erosion morphology of the electrically contacted materials; moreover, the potential mechanisms behind their arc erosion behavior were investigated in depth. The results demonstrate that the doping of rare earth elements can improve the electrical conductivity and physical properties of the contacts, optimize the arc parameters, and enhance their resistance to arc erosion. Notably, AgCuO-Ce exhibited the highest electrical conductivity and the least amount of material transfer; moreover, it had excellent arc time and energy parameters, resulting in the best resistance to arc erosion. This study provides a theoretical basis for the screening of doping elements to enhance the performance of AgCuO contact materials and offers new ideas and scientific references for this field.

## 1. Introduction

Electrical contacts are essential components in a wide range of electrical and electronic equipment and play a critical role in ensuring the stability, reliability, and longevity of electrical systems [1,2]. However, during their service life, electrical contacts are subject to various physical and chemical erosion processes such as high temperatures, welding, wear, and arc discharge [3,4,5]. As such, it is crucial for electrical contact materials to possess excellent resistance to arc erosion, good welding properties, and low contact resistance [6].

AgCuO electrical contacts are an environmentally friendly alternative to AgCdO electrical contacts, which are known to be toxic due to their cadmium content. Compared to AgSnO_2_ electrical contacts, AgCuO electrical contacts exhibit superior electrical conductivity. During electrical contact, the copper oxide in AgCuO partially decomposes to form a cuprous oxide, which possesses similar arc-extinguishing properties and exhibits good resistance to arc erosion that is akin to AgCdO. However, the arc-extinguishing properties of AgCuO are slightly inferior to those of AgCdO; thus, it is essential to improve the arc erosion resistance of AgCuO. Therefore, research focused on improving the arc erosion resistance of AgCuO is of great importance.

On the one hand, most research on AgCuO contact materials has focused on improving the preparation process, with little attention paid to the use of additives to enhance properties [7,8,9]. On the other hand, rare earth elements possess unique physical and chemical properties such as high melting points, high hardness, and good thermal stability. The addition of small amounts of rare earth elements can refine grain structures and significantly improve the mechanical properties and organization of the alloy [10]. Many researchers have successfully improved the electrical conductivity, arc erosion resistance, and wettability of electrical contact materials by adding rare earth elements. For example, Nguyen, T. H., et al. found that CuO is a narrow band gap semiconductor [11]. By combining their simulation with experimental studies, Zhang et al. found that the doping of rare earth elements (La, Ce, Y) could reduce the internal oxidation temperature of the contact material, refine the microstructure and grain size of the alloy, reduce the contact resistance and shorten the arc duration, enhance the arc erosion resistance of the contact, and improve the hardness of the alloy [12,13]. The doping of La in CuO refined the grain size and improved the stability and overall performance of CuO [14]. Zheng et al. found that the addition of rare earth elements to the contact material could produce oxides that could be suspended in the molten silver pool at high temperatures, thereby increasing the viscosity, reducing material transfer, and improving the electrical properties of the contact material [15].

In this study, cost-effective rare earth elements, namely, La, Ce, and Y, were chosen for the doping modification of the AgCuO contact materials. The physical properties and arc erosion resistance of the rare, earth-doped AgCuO electrical contact materials were investigated through a combination of first-principles calculations and experimental analyses. Firstly, the electronic structures and elastic constants of CuO and CuO-X (La, Ce, Y) were calculated using the first principle of density functional theory. The electrical conductivity and hardness of the doped systems were then obtained through computational analysis. For the results of electrical contact experiments, the variation of arc parameters such as arc duration, arc energy, welding force, contact resistance, and material transfer for the contact materials and the relationship between them were analyzed. The surface morphology of the eroded specimens was then analyzed using a 3D surface profiler and scanning electron microscopy, and the evolution mechanism of the arc erosion morphology was investigated. The results show that the rare earth elements La, Ce, and Y can enhance the arc erosion resistance of AgCuO contact materials to different degrees, among which Ce exhibits the most significant effect, which verifies the consistency between experimental results and theoretical calculations. This provides a new idea and a proven method for rare earth doping to improve the performance of AgCuO electrical contact materials and broaden their application scope.

## 2. Results and Discussion

During arc erosion, cyclic arc discharge, contact loading, and Joule heat will inevitably affect the surface condition of electrical contact materials, resulting in metal melting, sputtering, evaporation, oxidation, and, ultimately, the deterioration of the surface contact properties and performance of electrical contact materials. Therefore, studying the physical properties of electrical contact materials and evaluating the extent of erosion during arc erosion is of great theoretical and practical significance.

### 2.1. Analysis of Arc Parameters

#### 2.1.1. Arc Energy, Arc Duration, and Arc Stability

**Figure 1:**
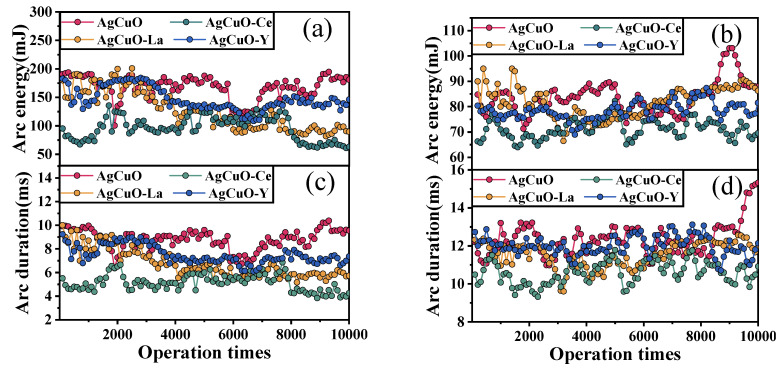
Average arc energy and arc duration along with contact operation times: (**a**,**c**) make arc, (**b**,**d**) break arc.

To gain a deeper understanding of the arc stability of various element-doped AgCuO contact materials, Figure 1 presents a graphical representation of the arc energy and arc duration as a function of the number of operations during arc making and arc breaking, respectively. Each data point is an average of 100 operations. It can be observed that both the arc energy and arc duration exhibit similar fluctuation trends. The rare earth element-doped contacts show reduced fluctuations in the contact material during both the make arc (Figure 1a,c) and break arc (Figure 1b,d); however, there is no significant trend for the arc duration and arc energy to increase with the number of operations. Moreover, in Figure 1a,c, it is evident that the undoped AgCuO contact material has a more pronounced increase in arc duration and arc energy, as the number of operations increases. Notably, the Ce-doped contact material demonstrates the shortest arc duration and the lowest arc energy. Furthermore, it can be deduced that the arc-making process exhibits less fluctuation in arc ignition time and arc energy compared to the arc-breaking process. Based on these results, it can be concluded that the AgCuO-Ce contact material possesses superior arc erosion resistance among the four materials tested.

#### 2.1.2. Welding Force Analysis

**Figure 2:**
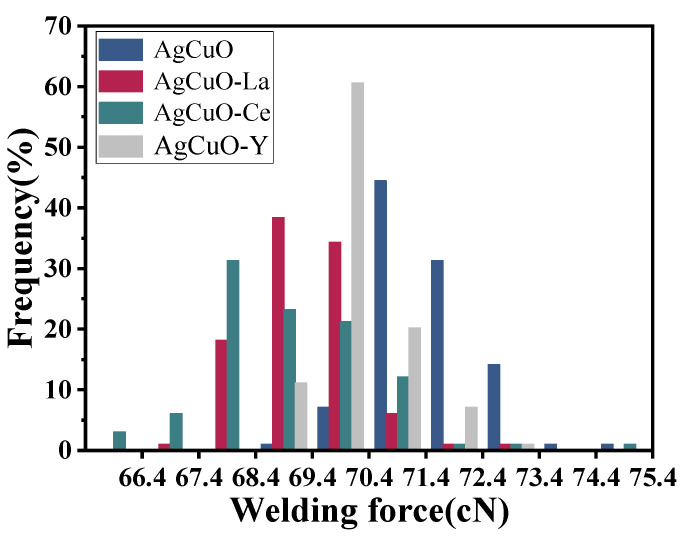
Welding force along with contact operation times.

The welding force magnitude and fluctuations can reflect a material’s resistance to welding, which occurs when the current passes through a closed contact point and causes contact melting and welding. Figure 2 illustrates the relationship between the welding force frequency and the welding force, where the height of each rectangle represents the frequency of the welding force within the corresponding interval. It is apparent that the undoped material exhibits a concentration of welding force in the higher value range, while the presence of La and Y dopants also shows a more focused and lower distribution compared to the undoped material. The Ce-doped material is relatively scattered in the lower regions, indicating a greater fluctuation in the welding force but a lower tendency to weld. Based on the aforementioned analysis, it can be concluded that rare earth-doped AgCuO contact material demonstrates improved resistance to melt soldering compared to the undoped material.

Contact welds are prone to arc erosion during their operation, which can cause the contact pair to fail. The formation of a contact weld relies on the arc energy and the tensile strength; hence, it is crucial to investigate the correlation between these parameters and the microstructure. By utilizing the welding force evaluation model, the welding force can be mathematically expressed as follows [16]:

(1) $$F_{W}=KW^{2/3}$$

where $F_{W}$ is the maximum welding force, K is the contact material factor, and W is the arc and Joule heating energy. As a rule, the arc energy is considerably higher than the Joule heating energy, which means that the total energy can be approximated by the arc energy, denoted as $W_{A}$. The coefficient K can be defined as follows:

(2) $$K=\Gamma \pi {\left[\frac{3}{4\pi \rho \left[C\left(T_{m}-T_{0}\right)+C_{L}\right]}\right]}^{2/3}$$

where Γ is the tensile strength of the material, ρ is the density of the material, C is the specific heat, $T_{m}$ is the melting temperature of the material, $T_{0}$ is the initial melting temperature, and $C_{L}$ is the latent heat of the melting of the material. For the four contact materials, there are no significant differences in relative density and specific heat. Therefore, it can be concluded from Equations (1) and (2) that the maximum welding force $F_{W}$ is mainly related to the tensile strength Γ and the arc energy $W_{A}$. The maximum welding force can therefore be expressed as

(3) $$F_{W}=C\Gamma W^{2/3}$$

where C represents the constant in the equation. It is evident that arc energy plays a significant role in determining the welding force. Therefore, the incorporation of rare earth elements to reduce the arc energy is crucial in minimizing the welding force, and the most significant outcome is obtained through doping with Ce elements.

#### 2.1.3. Relation of Arc Duration and Arc Energy

**Figure 3:**
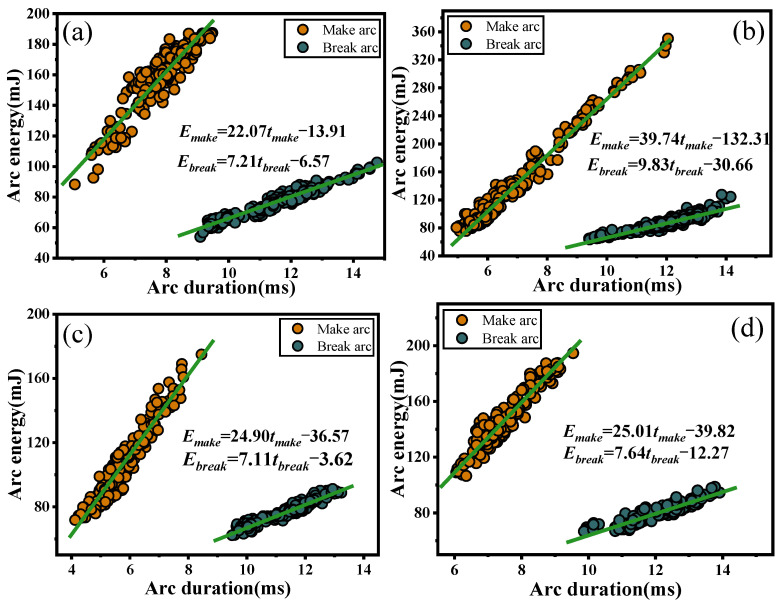
Variation of arc energy versus arc duration: (**a**) AgCuO, (**b**) AgCuO-La, (**c**) AgCuO-Ce, (**d**) AgCuO-Y.

To further understand the correlation between arc duration and arc energy, Figure 3 plots the arc duration and arc energy. It is evident that both arc duration and arc energy are linearly distributed, and fitting the data generates a linear relationship between arc duration and arc energy: *E_A_* = *k_A_·t_A_* + *b*, where *k_A_* is the material-related constant. From Figure 3, it is apparent that the rate of change of arc energy with arc duration increases after doping with rare earth elements and that the rate of change is minimal when Ce is the dopant element. Figure 3a reveals that some points deviate significantly from the fitted straight line due to the large amplitude of the oscillation of undoped AgCuO contacts and the poor stability of the arc energy. Comparing Figure 3a–d, it is apparent that doping with rare earth elements leads to a shorter arc duration and lower burning arc energy compared to the undoped material. Additionally, the arc energy of the break arc is lower than that of the make arc for a different arc duration, and the difference in energy between the two increases as the arc duration increases, which leads to a higher average arc power for the made arc than the broken arc.

#### 2.1.4. Distribution of Arc Duration and Arc Energy

**Figure 4:**
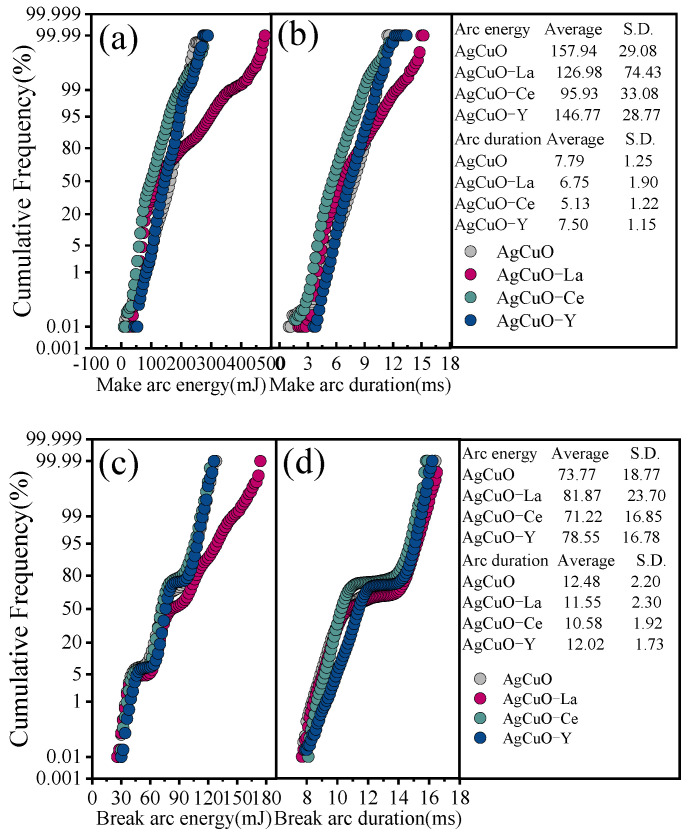
Distribution of arc duration and arc energy for the AgCuO contact material: (**a**,**b**) make arc, (**c**,**d**) break arc.

To better understand the influence of arc parameters on arc erosion in electrical contact tests, this can be investigated by analysing the recorded arc duration and the distribution, cumulative frequency, and trend of the arc energy [17]. The frequency ($P\left(x_{n}\right)$) statistics are performed using the number of bins of 100, and the cumulative frequency ($g\left(x_{n}\right)$) can be expressed as $g\left(x_{n}\right)=\overset{n}{\underset{1}{\sum }}P\left(x_{n}\right),\;n\in \left[1,\;100\right]$. Figure 4a,b shows the cumulative distribution of the make arc duration and make arc energy. In terms of cumulative distribution, the AgCuO-Ce contact material has the smallest average arc energy and arc duration, and the AgCuO-Y contact material has the smallest S.D. Figure 4c,d illustrates the cumulative distribution of arc break duration and arc break energy. Again, it can be seen that the AgCuO-Ce contact material has the smallest average value and that the AgCuO-Y contact material has the best stability.

#### 2.1.5. Analysis of Arc Voltage and Arc Current Waveform

**Figure 5:**
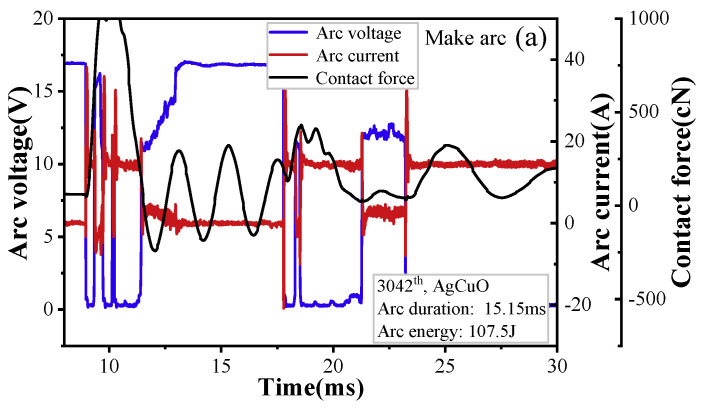

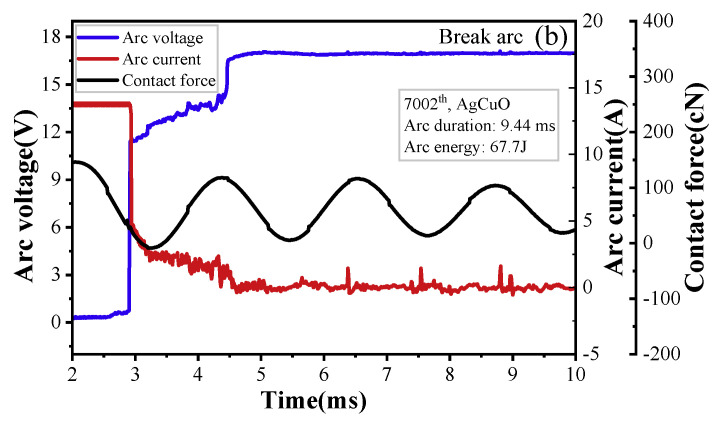
Typical arc current, arc voltage, and contact force waveforms: (**a**) make arc, (**b**) break arc.

To fully comprehend the inherent connection between arc parameters and surface topography, it is imperative to delve deeper into the fluctuations of arc voltage and arc current during electrical contact operations. Figure 5 displays the changes in arc voltage, arc current, and contact force over time. The stationary contact generates vibrations when the anode (moving contact) comes in contact with it during the arc-making process. The arc voltage and current vary with the contact force, which is proportional to the displacement.

The arc’s formation is dependent on the relative position of the moving and static contacts; therefore, the arc voltage increases with an increase in the pole gap, as shown in Figure 5a. Furthermore, it can be observed that the make arc is produced by the bouncing between the moving and static contacts and is extinguished when a reliable contact is established between the cathode and anode. In contrast, for the break arc, Figure 5b shows damped oscillations in the stationary contact. When compared to make arc, the break arc is initiated when the carrier density drops below a certain threshold, leading to an insufficient carrier with which to sustain the arc burning. As shown in Figure 5, the current of the break arc is significantly lower than that of make arc, resulting in lower arc power for the interrupted arc.

### 2.2. Material Transfer and Arc Erosion Morphology

**Figure 6:**
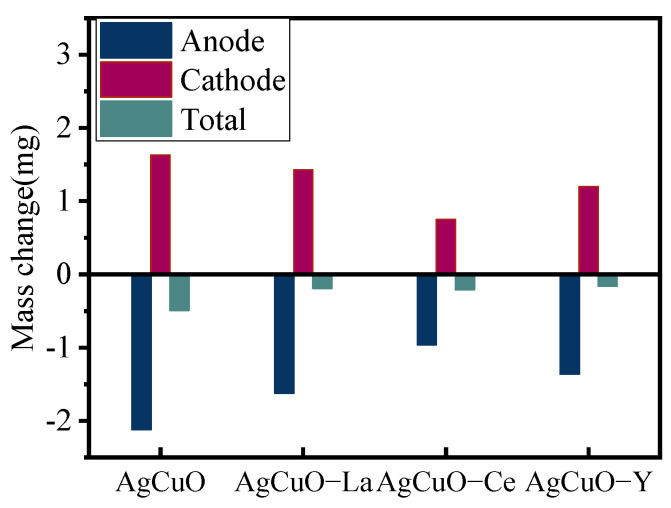
Mass change of the electrical contact material after 10,000 operations.

The transfer and loss of contact material due to arc erosion directly affects the life of electrical contacts and ultimately leads to component failure. When arc erosion occurs, the material separates from the electrode by vaporization, liquid splash, or detachment due to the application of the arc heat flux and arc forces to the electrode surface [18,19]. The change in quality of AgCuO electrical contact material after arc erosion testing is shown in Figure 6.

The cathode mass decreases and the anode mass increases after arc erosion. The mass transfer effect of undoped AgCuO material is significant, and, after doping with rare earth elements, both mass transfer and losses are reduced to varying degrees. The contact materials doped with Ce elements have a dense microstructure, uniform particle distribution, good mechanical and thermal properties, and suppress mass loss under erosion.

**Figure 7:**
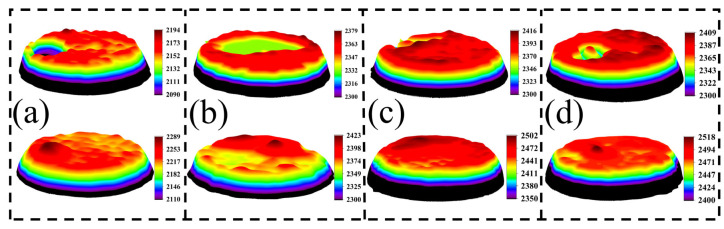
Three-dimensional macroscopic arc erosion morphology of the contacts after 10,000 operations: (**a**) AgCuO, (**b**) AgCuO-La, (**c**) AgCuO-Ce, (**d**) AgCuO-Y. Note: The top is the anode and the bottom is the cathode corresponding to it.

Figure 7 presents the three-dimensional macroscopic morphology of AgCuO contact material after 10,000 operations with different doping elements applied to the moving contact (anode) and the stationary contact (cathode). This morphology provides insights into the effects of arc erosion. The surface morphology of the material undergoes significant changes following arcing action. In the case of undoped AgCuO contact material (Figure 7a), the surface of the moving contact exhibits concentrated and large bulges, densely distributed small bulges, and several pits on the surface of the static contact. However, when rare earth elements are added (Figure 7b–d), the surface morphology changes. The La- and Ce-doped morphologies tend to have a dispersed appearance without excessive bulges and pits, while Y doping reduces the erosion area of the material. Incorporating rare earth elements into the material doping has been found to be effective in enhancing the erosion morphology of the contacts. Notably, Ce-doped material displays a flatter overall shape due to the refining effect of Ce on the material grain. This refinement leads to a more uniform distribution of CuO on the material surface, resulting in improved resistance to arc erosion.

**Figure 8:**
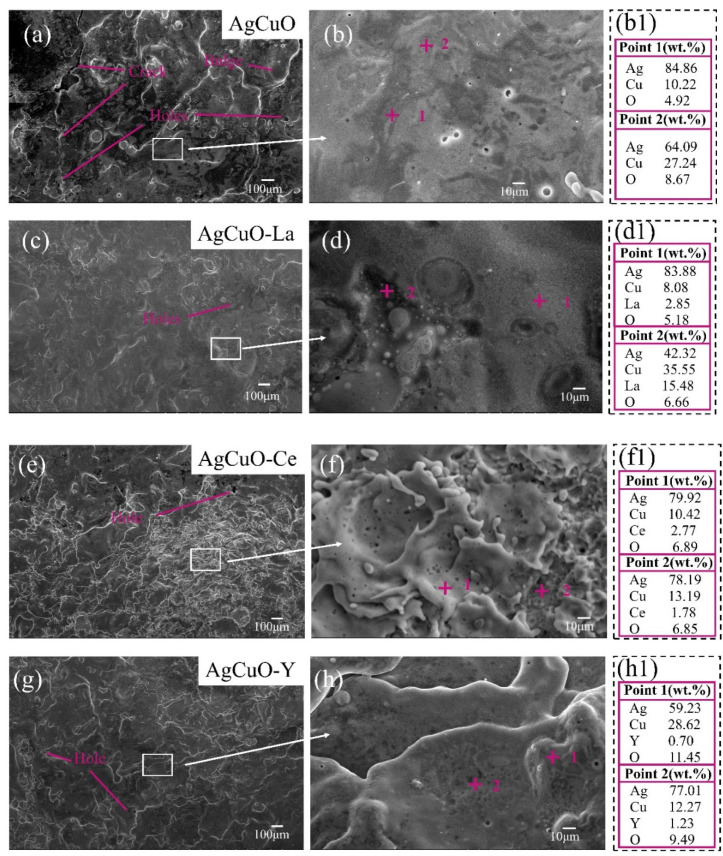
Surface morphologies of eroded cathode after 1000 operations: (**a**,**b**) AgCuO contact material at different magnifications, (**b1**) EDS results of the points marked in (**b**); (**c**,**d**) AgCuO-La contact material at different magnifications, (**d1**) EDS results of the points marked in (**d**); (**e**,**f**) AgCuO-Ce contact material at different magnifications, (**f1**) EDS results of the points marked in (**f**); (**g**,**h**) AgCuO-Y contact material at different magnifications, (**h1**) EDS results of the points marked in (**h**).

To investigate the impact of different doping elements on arc erosion, the microscopic morphology of the cathode contacts was analyzed using SEM, and the composition was examined using EDS. The SEM micrographs and EDS element mapping results are shown in Figure 8. Comparing the undoped contact surface at the same magnification (Figure 8a,c,e,g), it is evident that the surface exhibits numerous cracks, holes, and bulges, indicating poor morphology. However, after doping with rare earth elements, the microscopic morphology improves significantly. This improvement can be attributed to the addition of rare earth elements, promoting a more uniform material distribution after high-temperature arc melting. In Figure 8b, the gray bulges correspond to aggregated areas of silver, as confirmed by the EDS element mapping at point 1, while the dark areas within the bulges, like point 2, represent aggregated areas of copper. The uneven distribution of silver and copper oxide in the undoped contact material contributes to the observed variations. After La doping (Figure 8c), the material surface becomes noticeably flatter with minimal cracks and only a few small holes. A partial enlargement (Figure 8d) reveals that the surface after La doping consists mostly of uniformly mixed areas, similar to point 1. However, the area around point 2 exhibits a poorer surface condition due to the aggregation of Ce and La. The presence of Ce elements (Figure 8e) facilitates grain refinement, resulting in a finer eroded surface phase. This refinement helps prevent the formation of large holes and cracks, thereby improving the erosion morphology. A local enlargement (Figure 8f) further emphasizes this improvement. The eroded surface of AgCuO-Y (Figure 8g) appears generally more uniform, with some small holes. The highlighted bulge at point 1 in the local enlargement (Figure 8h) is caused by the agglomeration of copper.

The addition of rare earth elements to the contact material significantly improved the micromorphology of the erosion surface. This improvement resulted in a more uniform appearance with a reduced presence of cracks, holes, and bulges. The beneficial effect of rare earth elements can be attributed to their ability to refine the grain structure of the material. This refinement led to a more uniform distribution of CuO within the silver matrix, thereby minimizing surface erosion caused by the arc.

### 2.3. Arcing and Welding Processes and the Evolution Mechanism of Arc-Eroded Morphology

**Figure 9:**
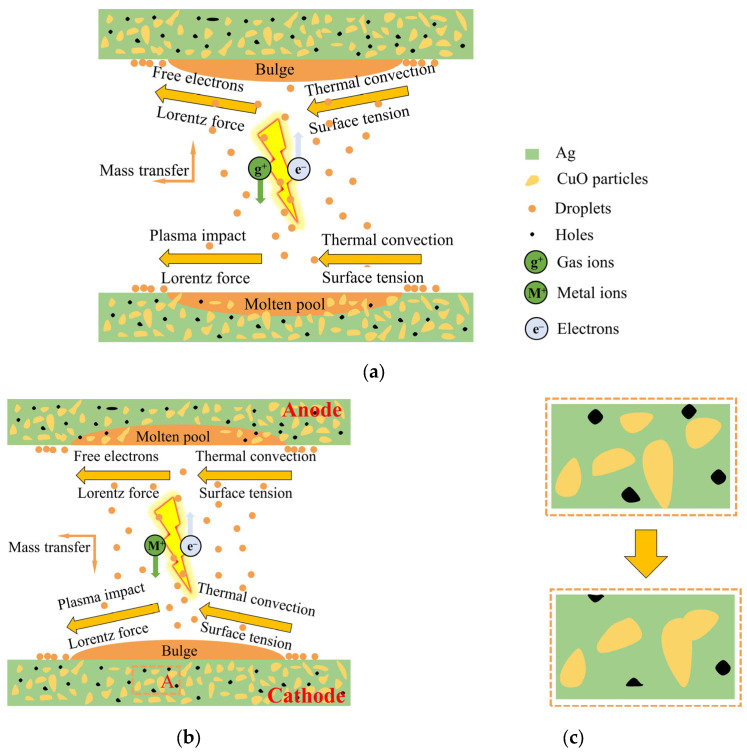
Schematics of (**a**) cathode-to-anode material transfer, (**b**) anode-to-cathode material transfer, (**c**) the change in morphology of the selected area A before and after the test in Figure 9b.

Studying the material transfer process provides valuable insights into the mechanism of contact material surface failure and transfer, which greatly impacts the surface morphology of the contacts. Figure 9 illustrates this process of change. The arc discharge process can be divided into two phases: the gas phase and the metal phase [20,21]. In Figure 9a, the gas phase involves the ionization of air between the contacts, generating gaseous cations that are rapidly propelled by the electric field towards the cathode. This creates a powerful plasma shock, inducing flow in the central region of the cathode. Simultaneously, the Lorentz force resulting from the electric current drives the molten material toward the sidewalls, causing splashing. However, thermal convection and surface tension limit the inward flow of the molten metal from the sides of the pool. The main factors causing splashing are the impact of the plasma and the Lorentz force, while thermal convection and surface tension primarily prevent it. The anode experiences similar forces as the arc-eroded cathode, but the bombardment of free electrons plays a dominant role in mass transfer to the anode. As a result, the effect on the anode is less pronounced compared to the cathode, resulting in rare instances of sputtering. Sputtered particles mainly originate from the cathode and are transferred to the anode, where they redeposit on its surface, known as cathode–anode transfer [20].

During the metal phase, as the contacts separate, a molten bridge forms, facilitating mass transfer between the cathode and the anode [22]. The high-temperature region near the cathode results in material transfer from the bridge to the cathode surface upon fracture. Metal or metal oxide vapors, with lower ionization energy than air, preferentially enter and undergo ionization within the arc, altering the plasma composition. The anode undergoes electron bombardment, causing the evaporation of anode material and filling the contact gap with metal vapor. The highly conductive metal vapor drives the arc, with the generated metal ions being accelerated towards the cathode and deposited on its surface, as depicted in Figure 9b. In AgCuO contact materials, the metal phase arc dominates the electrical contact process, leading to cathode bulging and the presence of etch pits and spray drops on the anode surface.

During the melting process, the low density of CuO particles causes them to gradually rise within the Ag pool, leading to the local surface aggregation of Ag and CuO. This results in the formation of holes and a rough surface in the Ag- and CuO-enriched areas. However, the increasing mobility of the AgCuO contact material during the on–off test led to a more homogeneous mixture [23]. Consequently, the number of holes slightly decreases, as shown in Figure 9c. The evolution of micromorphology with the number of operations plays a crucial role in the observed decrease in arc energy and arc duration, as depicted in Figure 1. In the case of the AgCuO contacts doped with rare earth elements, the erosion of the contact surface becomes more uniform, and the rare earth metal dopant is evenly dispersed throughout the matrix. This dispersion stabilizes the melt pool by increasing the viscosity of the liquid Ag and CuO, thereby inhibiting liquid splash and reducing erosion. The introduction of doping promotes arc dispersion, leading to lower arc energy and reduced erosion. It also decreases the likelihood of large arc pits and arc craters formation. When the arc is extinguished and the molten metal pool rapidly solidifies, the doped CuO remains uniformly distributed in the Ag matrix. This reduces the formation of Ag- or CuO-enriched areas, ensures that the electrical contact characteristics of the contact material do not deteriorate, and enhances its resistance to arc erosion.

## 3. First-Principles Calculation and Discussion

### 3.1. Calculation Models and Methods

The heat generation and elastic constants of the components in contact materials were calculated using the Cambridge Series Total Energy Package (CASTEP) based on the density functional theory (DFT) [24]. This simulation method is widely used for the calculation of materials with periodic structures such as ceramics, semiconductors, and metals.

In the generalized gradient approximation (GGA) method, the exchange–correlation function is modified in the form of Perdew–Burke–Ernzerhof (PBE) [25]. This approximation method overcomes the drawback of describing the real system in the case of drastic changes in density and improves the calculation of the exchange–correlation energy. The OTFG supercomplex pseudopotential formulation with an inverse easy space is used to calculate the potential function [26]. The BFGS conjugate gradient algorithm was used for optimization [27]. After convergence tests, the plane wave truncation energy value was taken as 500 eV, and the spatial grid points were set as 5 × 7 × 9. The convergence accuracy was 1.0 × 10^−6^ eV/atom, the residual force per atom was less than 0.1 eV/nm, the tolerance displacement was less than 5.0 × 10^−4^ nm, and the stress deviation was less than 0.02 GPa.

**Figure 10:**
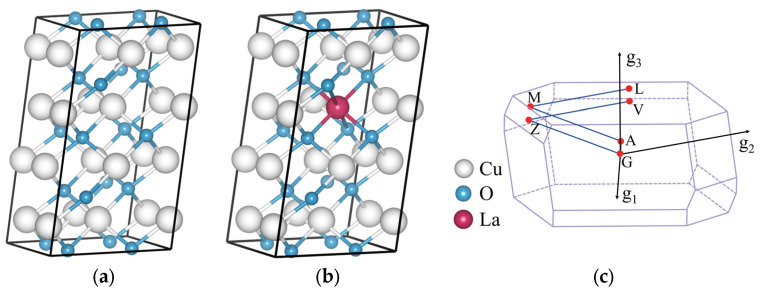
Crystal structures of a 2 × 2 × 1 supercell with (**a**) CuO, (**b**) La-doped CuO. (**c**) The associated Brillouin zone.

The CuO 2 × 2 × 1 supercell model was constructed as shown in Figure 10a. The doping model utilized atomic substitution to replace Cu atoms in the crystal cell (Fractional XYZ = 0.625, 0.375, 0.5), ensuring that the atomic doping ratio was maintained at 6.25%. An example of the doped CuO supercell model with La doping is presented in Figure 10b, while Figure 10c displays the corresponding Brillouin zone and calculation paths.

### 3.2. Crystal Parameters and Stability Analysis

To ensure the reliability and accuracy of crystal electronic structure analysis, energy calculations, and elastic constant calculations, the optimization process begins with CuO and rare, earth-doped CuO. An iterative process is used to adjust the atomic coordinates and cell parameters, minimizing energy and identifying the most stable point of the structure that is closest to reality.

The enthalpy of formation is the amount of energy released or absorbed by different types of atoms from the ground state into a compound, and it indicates the ease of the formation of the compound. Equation (4) is the formula for the enthalpy of formation [28].

(4) $$\Delta H=\frac{E_{tot}^{AB}-N_{A}E_{solid}^{A}-N_{B}E_{solid}^{B}}{N_{A}+N_{B}}$$

where $E_{tot}^{AB}$ is the total energy of the crystal after optimization; $E_{solid}^{A}$ and $E_{solid}^{B}$ are the average energy of each atom of solid elements (*A* and *B*, respectively); $N_{A}$ and $N_{B}$ are the numbers of *A* and *B* atoms respectively.

In the case of a negative enthalpy of formation, the larger the absolute value, the more stable the intermetallic compound. Table 1 shows the optimized lattice constants, volumes, and enthalpies of formation. Both CuO and rare, earth-doped CuO exhibit negative enthalpies of formation, indicating a stable structure. The doping model is more stable, with varying degrees of volume increase after doping with different rare earth elements.

### 3.3. Electronic Properties

**Figure 11:**
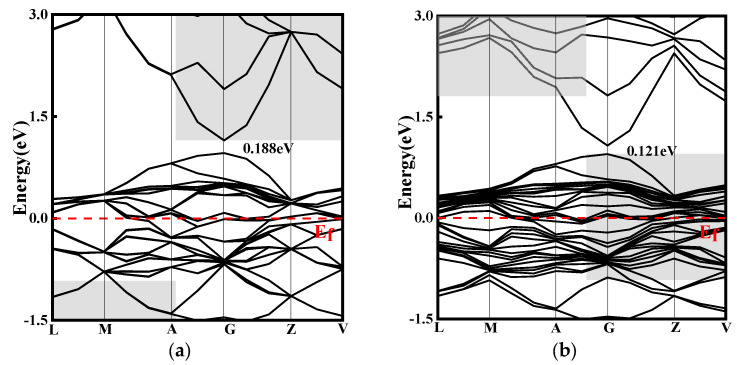

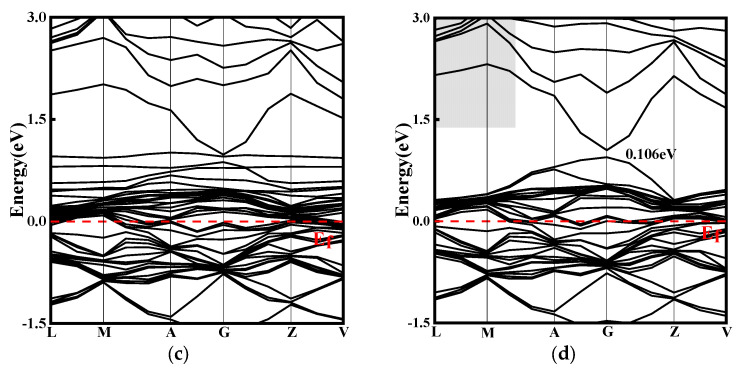
Band structure: (**a**) CuO. (**b**) La-doped. (**c**) Ce-doped. (**d**) Y-doped.

To investigate the effect of doping on the electronic structure of CuO, energy band structure calculations were performed, and the results of this are presented in Figure 11. The simulated band gaps for CuO, CuO-La, CuO-Ce, and CuO-Y were 0.188 eV, 0.121 eV, 0 eV, and 0.106 eV, respectively. These values are smaller than the experimental band gap values of 1.2~2.1 eV for CuO [29]. This discrepancy can be attributed to the first principles and the generalized gradient approximation used in the calculations, which overestimate the energy of Cu atoms and enhance the hybridization between Cu and O orbitals, resulting in a broadening of the valence band and a smaller calculated energy gap value. Nevertheless, the simulation method and parameters are consistent, and the focus of this paper is to compare the evolution of the band gap before and after doping.

Figure 11 shows that doping CuO with rare earth elements reduces its band gap, resulting in denser conduction and valence bands. As a result, more electrons are transferred from the valence band to the conduction band, leading to an improvement in electrical conductivity. The introduction of Ce elements by doping creates several new energy bands near the Fermi level, resulting in the smallest band gap and the most significant improvement in electrical conductivity. In addition, the doping of La, Ce, and Y elements leads to denser energy bands in both the conduction and valence bands, indicating stronger atomic interactions.

**Figure 12:**
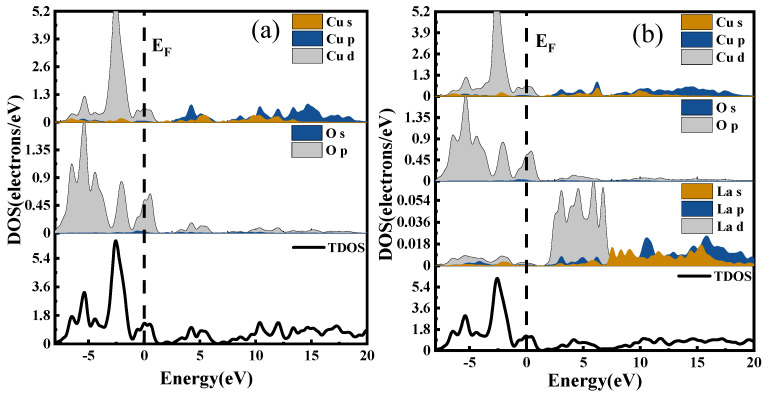

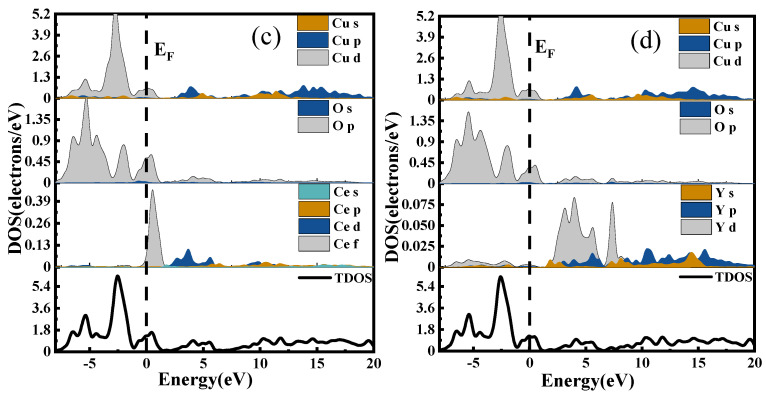
Densities of states: (**a**) CuO, (**b**) La doped, (**c**) Ce doped, (**d**) Y doped.

The density of states is a way to visualize the energy bands that are more intuitive than the energy band structure itself. It represents the distribution of electrons at different energy levels, that is, the number of electron states per unit energy interval in the system. Different atomic orbitals have different energies; therefore, the density of states can be used to characterize the arrangement of electrons outside the nucleus and the interactions between atoms [30]. The Total Density of States (TDOS) represents the energy distribution of all electrons in the system, while the Partial Density of States (PDOS) indicates the bonding of electrons in different orbitals. The DOS of CuO and CuO-X (La, Ce, Y) are shown in Figure 12a, in which the densities of states of CuO, (b), (c), and (d) are the densities of states of CuO doped with La, Ce, and Y, respectively.

In the valence band region near the Fermi level, the d electronic state of La hybridizes with the p electronic states of Cu and O, resulting in an increase in carrier number and an enhancement in electrical properties for the La-doped system compared to the undoped system.

When Ce is doped into the CuO system, the s and p electronic states of Cu in the conduction band region overlap with Ce’s electronic states, leading to enhanced electronic interactions. Consequently, the conduction band shifts significantly towards lower energy levels. As electrons at absolute zero temperature fill all energy levels below the Fermi level, the Fermi surface separates the occupied and unoccupied states, with electrons near the Fermi level being most likely to transition. Therefore, the electrical properties of metallic materials are largely determined by electronic states near the Fermi level. The Ce doping results in clear f orbital contribution near the Fermi level, which plays a crucial role in improving electrical conductivity.

The doping of the Y element induces the hybridization of its d electronic state with the p electronic states of Cu and O, leading to an increase in carrier number. Additionally, the s and p electronic states of Cu overlap with the region of action in the conduction band, enhancing their degree of action and causing a considerable shift of the conduction band towards lower energy levels. As a result, the electrical conductivity of the Y-doped system is improved.

After analyzing the energy band structure and density of states, it can be concluded that doping with all three rare earth elements (La, Ce, and Y) enhances the electrical conductivity of CuO.

### 3.4. Elastic Constant

According to Hooke’s law, the elastic constants depend on the stress and strain tensor. The elastic constant $C_{ijkl}$ can be written as [31,32,33]:

(5) $$C_{ijkl}={\left(\frac{\partial \sigma_{ij}\left(x\right)}{\partial e_{kl}}\right)}_{X}$$

where $e_{kl}$, $\sigma_{ij}$, X, and x are the Eulerian strain tensor, the applied stress tensor, and the coordinates before and after deformation, respectively. For the tetragonal crystals studied in this paper, six independent elastic constants *C*_11_, *C*_33_, *C*_44_, *C*_66_, *C*_12_, and *C*_13_ can be obtained. The bulk modulus (*B*) and the shear modulus (*G*) are derived from the elastic constants. Based on the Voigt and Reuss method [34], for tetragonal crystals, the bulk modulus and shear modulus are defined as

(6) $$B_{V}=\frac{2C_{11}+2C_{12}+4C_{13}+C_{33}}{9}$$

(7) $$B_{R}=\frac{1}{2\left(S_{11}+S_{12}\right)+S_{33}+4S_{13}}$$

(8) $$G_{V}=\frac{2C_{11}-C_{12}-2C_{13}+C_{33}+6C_{44}+3C_{66}}{15}$$

(9) $$G_{R}=\frac{15}{8S_{11}-4S_{12}-8S_{13}+4S_{33}+6S_{44}+3S_{66}}$$

where $S_{ij}$ is the flexibility factor, $S_{ij}=C_{ij}^{-1}$. The Voigt–Reuss–Hill (VRH) mean, which is the arithmetic mean of the Voigt and Reuss bounds, is generally considered to provide the most accurate estimate of the isotropic modulus of elasticity. Using the VRH average, the bulk modulus and shear modulus are $B=\left(B_{R}+B_{V}\right)/2$ and $G=\left(G_{R}+G_{V}\right)/2$, respectively. The average value of *E* and Poisson’s ratio (*v*) can be expressed in terms of *B* and *G* as follows [35].

(10) $$E=\frac{9BG}{3B+G}$$

(11) $$v=\frac{3B-E}{6B}$$

If the elastic constants satisfy the Born stability criterion, the crystal structure is usually considered to be mechanically stable [36,37]. The positive determinant of the crystal symmetry matrix is a necessary condition for the crystal stability criterion. For a tetragonal crystal, the mechanical stability criterion can be described as

*C*_11_ > 0, *C*_33_ > 0, *C*_44_ > 0, *C*_66_ > 0,

(*C*_11_ − *C*_12_) > 0, (*C*_11_ + *C*_33_ − 2*C*_13_) > 0,

[2(*C*_11_ + *C*_12_) + *C*_33_ + 4*C*_13_] > 0.

All of the elastic constants meet the Born stability criterion, suggesting that the structure is stable both prior to and following doping.

Currently, a combination of resistance, bond strength, and electronegativity models can be used to evaluate the hardness of known crystals, making it feasible to predict the hardness based on the designed crystal structure using these models. Using this approach, fitting corrections were made to the data set of Chen et al. [38], resulting in hardness equations containing *B* and *G* [39].

(12) $$H_{V}=0.9{\left(G/B\right)}^{1.137}G^{0.708}$$

The simulated hardness values obtained from the calculations are presented in Table 2. The La-doped samples exhibit the highest hardness, implying good wear resistance, while the Ce-doped samples demonstrate good toughness.

## 4. Experimental

### 4.1. Materials Preparation

The conventional powder metallurgy method can only achieve mechanical mixing of doped element powder, CuO powder, and silver powder, which makes it difficult to incorporate doped elements into the CuO lattice, thus deviating from the theoretical model presented in this paper. To ensure that the experimental results are more convincing and comparable to the CuO model with different doping elements obtained using the atomic substitution method, various rare earth element-doped nano-CuO powders were prepared using the sol–gel method.

Inorganic salts (MCl_n_) serve as precursors in the sol–gel method. Ammonia is gradually added dropwise to the solution to promote the hydrolysis reaction and the formation of a chemical precipitate. During hydrolysis, the main reactions can be expressed as M^m+^ + mH_2_O → M(OH)_m_ ↓ + mH^+^ [40]. The gel was obtained via sufficient washing and precipitation through filtration, with the main reactions being: M(OH)_m_ ↓ → MO_m/2_ + m/2H_2_O [41]. The colloids were eventually subjected to drying and sintering to produce a powder. The primary components and their respective ratios required for the preparation of rare earth element-doped CuO nanopowders are presented in Table 3, with the material ratios determined based on a 6.25% atomic doping ratio in accordance with the cell model.

To confirm that the sol–gel prepared doped CuO corresponds to the replacement cell model established by the simulation, the physical phase structure of the powder was analyzed using X-ray diffractometry. Y-doped CuO powder was used as an example of the doping structure. The diffractometer had a wavelength of 0.15405 nm, a scanning range of 6° to 90°, and a scanning speed of 5°/min.

**Figure 13:**
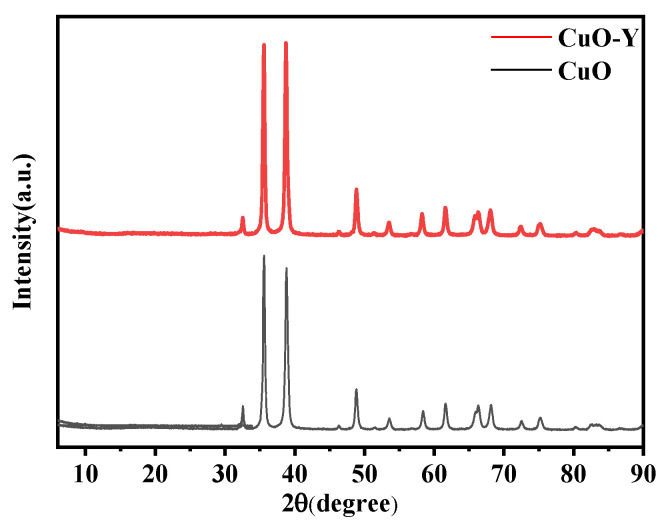
X-ray diffraction pattern.

The X-ray diffraction patterns of undoped CuO and Y-doped CuO are presented in Figure 13. As can be observed from the figure, the peaks of Y-doped CuO are essentially identical to those of pure CuO, with enhanced diffraction peaks. This suggests that the crystal structure of CuO remains unaltered after doping and that no new phases are formed. In other words, during the sol–gel synthesis process, the doped elements were incorporated into the CuO solid solution, which is consistent with the simulation model.

**Figure 14:**
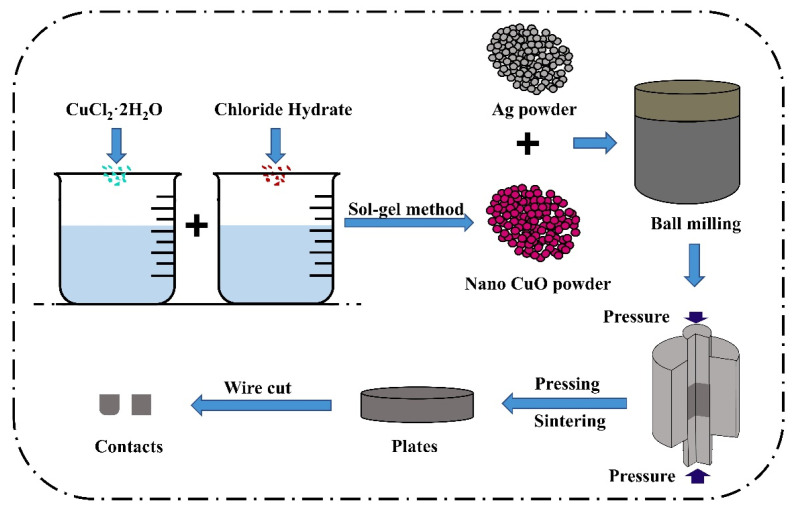
Schematic diagram of the preparation process of rare earth metal-doped AgCuO contacts.

High-energy ball milling and powder metallurgy techniques were used to prepare the aforementioned powders into uniformly structured and compositionally distributed doped AgCuO contact material, thereby overcoming the limitations of the powder metallurgy method, such as non-uniform mixing and powder agglomeration. In this experiment, a 10 g sample of AgCuO contacts was prepared, with a Ag to CuO ratio of 85:15. To create the doped AgCuO contacts, 8.5 g of Ag powder and 1.5 g of CuO-X (La, Ce, Y) powder, prepared using the sol–gel method, were ground together using a high-energy ball mill. This ensured that the doped CuO powder and Ag powder were evenly mixed. The mixture was then subjected to primary pressing, primary firing, re-pressing, re-firing, polishing, and cutting to produce contact specimens with a diameter of 3.2 mm and a thickness of 3 mm. Figure 14 illustrates the preparation process.

### 4.2. Density, Conductivity, and Hardness Testing

After the preparation of the material, its density, hardness, conductivity, and average contact resistance were measured, as indicated in Table 4.

The density of the contact materials was determined using the Archimedean drainage method, with measurements taken using distilled water. The measurement error was ±0.01 mg, and the weighing accuracy was ±0.001%. The mass of the sample in air was denoted as *m*_0_, and the mass in distilled water was denoted as *m*_1_. The density equation used is as follows.

(13) $$\rho =\frac{m_{0}}{m_{0}-m_{1}}\cdot \rho_{water}$$

where $\rho_{water}$ is the density of distilled water.

Although distilled water exhibits varying densities at different temperatures, the discrepancies are negligible. Five measurements were taken for each sample, and the average of the five measurements was taken.

The hardness of the contact material was gauged through the employment of an HXD-1000TM digital micro hardness tester. Five positions were selected for each sample, five individual hardness values were read, and then the average of the five sets of data was taken, as shown in Table 4.

The conductivity of the contact material was determined using an eddy current-based SIGMASCOPE SMP10 metal conductivity tester. Each specimen was measured five times, and the results were averaged and listed in Table 4. The data revealed that the conductivity of the material was enhanced by the addition of rare earth elements. This improvement was attributed to the positive impact of the rare earth elements on the material’s microstructure. Among the rare earth elements, Ce had the most significant effect on improving conductivity.

The density of a material is a crucial indicator of its compactness. A lower density implies that the material has more internal porosity. When contact interruption occurs in a contact, the ablation resistance of the contact material decreases, and the contact resistance increases, which has a severe impact on the electrical performance of the contact. As shown in Table 4, the density of the AgCuO contact material increased after doping with rare earths, with AgCuO-Ce exhibiting the highest density, followed by AgCuO-La, which is consistent with the findings on electrical conductivity. Moreover, the average contact resistance also shows a tendency to decrease, and its trend is consistent with the results of electrical conductivity.

### 4.3. Electrical Contact Test

**Figure 15:**
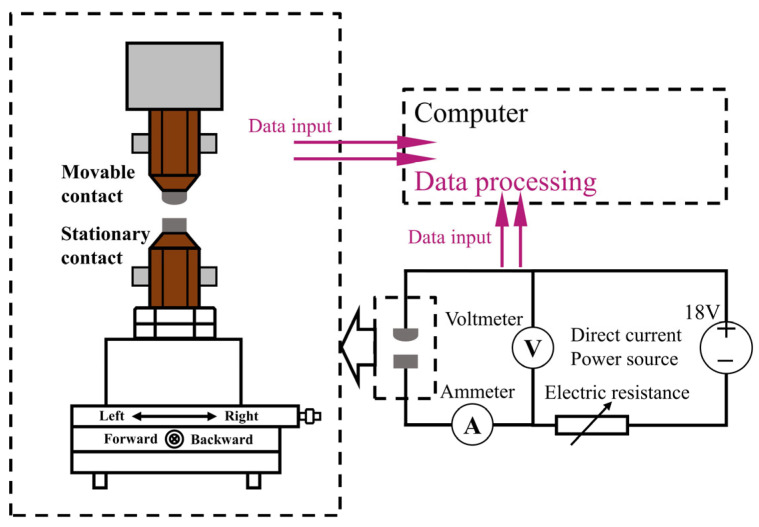
Schematic diagram of JF04D electrical contact testing system.

The JF04D electrical contact test system was utilized to conduct the electrical contact arc erosion test, the principle of which is depicted in Figure 15. The test system can emulate the actual operation of the contact, with the active contact serving as the anode contact and the stationary contact as the cathode contact. The experimental conditions are detailed in Table 5, and the device can gather data on arc time, arc energy, and welding force.

Upon completing the arc erosion test on the electrical contacts, the alteration in mass was ascertained by employing a Sartorius Genius series ME235S electronic analytical balance, which is capable of precisely measuring masses exceeding 0.01mg. Additionally, the three-dimensional morphology was evaluated using a three-dimensional profiler. The microscopic morphology was analyzed using scanning electron microscopy (SEM, Hitachi S-4800), and the composition was examined using energy-dispersive X-ray spectroscopy (EDS).

## 5. Conclusions

In this study, we investigated the stability, electronic structure, and elastic constants of undoped CuO as well as CuO doped with La, Ce, and Y using first-principles calculation. Doped CuO nanopowders were then synthesized using the sol–gel method, and their structures were confirmed via X-ray diffraction analysis to be consistent with the established rare earth-doped CuO cell model. AgCuO contacts, both undoped and doped, were prepared using the powder metallurgy method, and electrical contact experiments were conducted to analyze the arc parameters, arc erosion, and erosion mechanism of the contacts. The findings of this research demonstrate that:

(1) The simulation results demonstrate that the stability, electrical conductivity, and physical properties of the doped materials are improved to some degree. Specifically, the Ce-doped material exhibits the highest electrical conductivity, while La-doping enhances the material’s hardness. Conversely, the hardness of Ce- and Y-doped materials is reduced; however, their toughness is enhanced;

(2) It is demonstrated that the addition of rare earth elements significantly affects the arc behavior of the contact material. Specifically, rare earth doping enhanced the electrical conductivity of the contact material, which is consistent with the simulation results, while reducing the transfer and contact resistance of the material, shortening the arc duration and reducing the arc energy, smoothing the arc gradually, and reducing the welding force. SEM results showed that the doping of rare earth elements reduced the cracks, holes, and bulges on the material surface and significantly reduced arc erosion. Overall, the addition of rare earth elements improved the physical and electrical properties of the contact material and enhanced its resistance to arc erosion;

(3) The most significant doping effect of the cerium (Ce) element was observed in low-concentration doping conditions. This doping resulted in the most substantial increase in the electrical conductivity of the contact material; moreover, the average arc duration and arc energy were minimized. In addition, the erosion of the material surface was more uniform, without obvious cracks and holes, and the mass transfer and mass loss are relatively small. Meanwhile, the surface morphology of the material after doping with the Ce element was relatively flat. Therefore, the Ce element showed the best enhancement effect on the resistance to arc erosion of AgCuO contact materials.

## Figures and Tables

**Table 1:** Optimized cell parameters.

Model	Lattice Constant			Volume/Å^3^	Enthalpy/eV
	a/Å	b/Å	c/Å		
CuO	8.3802	8.3802	10.2360	718.6620	−0.9075
CuO-La	8.4256	8.4256	10.3347	733.6120	−11.9685
CuO-Ce	8.4135	8.4135	10.3589	733.2270	−0.9836
CuO-Y	8.4033	8.4033	10.3196	728.7140	−0.9850

**Table 2:** Bulk modulus B, shear modulus G, elastic modulus E, and hardness of rare metal-doped CuO.

	B/GPa	G/GPa	E/GPa	HV/GPa
CuO	136.53	28.43	117.58	1.09
CuO-La	130.66	22.04	62.59	1.65
CuO-Ce	134.05	11.60	33.83	0.32
CuO-Y	127.77	19.80	56.49	0.91

**Table 3:** Main raw material ratio.

Material	CuCl_2_·2H_2_O: LaCl_3_·7H_2_O	CuCl_2_·2H_2_O: CeCl_3_·7H_2_O	CuCl_2_·2H_2_O: YCl_3_·6H_2_O
Atomic ratio	93.75:6.25	93.75:6.25	93.75:6.25
Mass ratio	6.84:1	6.83:1	8.38:1

**Table 4:** Density, hardness, and electrical conductivity of the four contact materials.

	Density/g·cm^−3^	Hardness/HV	Conductivity/IACS%	Average Contact Resistance/mΩ
AgCuO	9.24	87.13	58.60	5.8144
AgCuO-La	9.41	89.50	60.78	2.0746
AgCuO-Ce	9.74	85.17	64.64	2.0240
AgCuO-Y	9.34	86.53	60.76	2.6560

**Table 5:** Parameters of arc erosion test conditions.

Parameter	Value
Frequency	0.5 Hz (On 0.5 s)
Operation number	10,000
Switching mode	DC mode
Contact force	86 cN
Surrounding gas	Air
Electrode spacing	2 mm
Circuit condition	DC18 V, 15 A

## Data Availability

The data presented in this study are available upon request from the corresponding authors.
